# Comparative study of Normal-phase versus reversed-phase HPTLC methods for the concurrent quantification of three antiviral agents against COVID19: Remdesivir, favipiravir and Molnupiravir: trichromatic sustainability assessment

**DOI:** 10.1186/s13065-025-01439-9

**Published:** 2025-03-28

**Authors:** Dina Salah El-Kafrawy, Amira H. Abo-Gharam

**Affiliations:** Pharmaceutical Chemistry Department, Faculty of Pharmacy, Alexandria University, Elmessalah, Alexandria 21521 Egypt

**Keywords:** Normal-phase HPTLC, Reverse-phase HPTLC, Three anti-Covid19 agents, Greenness, Blueness, Whiteness

## Abstract

The pursuit of sustainability in analytical chemistry is a multifaceted, challenging and complex endeavor. This requires continuous and competitive attempts to achieve the sustainable development goals at every step of the analytical methodology by adhering to the principles of green, blue and white analytical chemistry. This also involves assessment of the degree of sustainability using the latest evaluation metrics until finally reaching the design of a trichromatic procedure. The herein illustrated work represents a comparative study between two newly developed normal-phase and reverse-phase HPTLC methods for the simultaneous quantitative determination of Remdesivir (RMD), Favipiravir (FAV) and Molnupiravir (MOL). For normal-phase HPTLC method, the employed mobile phase consisted of ethyl acetate: ethanol: water (9.4:0.4:0.25, v/v), while, for reverse-phase HPTLC procedure, a greener mobile phase was employed consisting of ethanol: water (6:4, v/v). For both methods, detection wavelength of RMD and MOL was 244 nm while FAV was detected at 325 nm. Both methods were validated following the International Council for Harmonisation (ICH) guidelines with respect to linearity, range, accuracy, precision and robustness. The two established methods were proved to be linear over the range of 50-2000 ng/band for FAV and MOL and over the range of 30–800 ng/band for RMD. The excellent linearities were proved by the high values of correlation coefficients not less than 0.99988. The developed methods were successfully applied for the determination of the three drugs in their bulk form and in their pharmaceutical formulations. Furthermore, a thorough comparative and integrative trichromatic evaluation of sustainability of the designed methods was performed. The Analytical Eco-Scale, the novel Modified Green Analytical Procedure Index (MoGAPI) (2024) and the Analytical GREEnness (AGREE) metrics were applied for greenness assessment and the recent Blue Applicability Grade Index (BAGI) (2023) tool was employed for blueness evaluation. Finally, the RGB12 model was implemented for appraisal of whiteness of the developed methods.

**Supplementary Information:**

The online version contains supplementary material available at 10.1186/s13065-025-01439-9.

## Clinical trial number

Not applicable.

## Introduction

Human continuous erroneous attempts in the pursuit of tremendous industrial development have led to progressive environmental pollution and terrifying climate changes. This in turn requires humanity to strive to follow the 2030’s Agenda for “Sustainable Development” of the United Nations and to take into consideration achieving its goals in various fields including analytical chemistry. Recently, White analytical chemistry (WAC) represents the state-of-the-art paradigm in sustainable analytical chemistry. Preceded by green analytical chemistry (GAC) concept and its principles, WAC adopts 12 principles pertaining to 3 main pillars: analytical performance, eco-compatibility and practicality, thus representing a more insightful and scrupulous alternative to GAC principles [1]. Lately, the novel BAGI (Blue Applicability Grade Index) metric [2] focuses on assessing the analytical method’s practicality and applicability representing a complementary blue concept to WAC complying with the fit-for-purpose viewpoint. Implementation of trichromatic (green, blue and white) analytical techniques in pharmaceutical quality control laboratories would be the result of fruitful cooperation between analytical chemistry research centers and drug manufacturing companies. This beneficial partnership helps in tackling economical, practical and many ecological issues and in meeting the sustainable development goals in drug industry.

Since 2019, the Covid-19 pandemic has attacked the population worldwide. The Covid-19 virus infects the respiratory system causing a severe syndrome that may harm diverse body organs such as kidneys, liver, muscles, and the nervous system as well [3]. From this date, the accelerated development and approval of anti-SARS-CoV-2 vaccines helped in attaining some relief. Nevertheless, due to the insufficient vaccination of peoples worldwide and the individual varieties in immunization along with the rise of SARS-CoV-2 variants and continuous decline in antibodies concentrations in vaccinated humans with time, there is still an ongoing need for effective anti-Covid-19 agents. This urgent need during the pandemic spread has prompted the researchers to work on repurposing the already approved antiviral agents for use as anti-Covid-19 treatments. So far, three promising antiviral agents were found efficacious as anti-SARS-CoV2 agents namely, remdesivir (RMD), favipiravir (FAV) and molnupiravir (MOL).

**Fig. 1:**
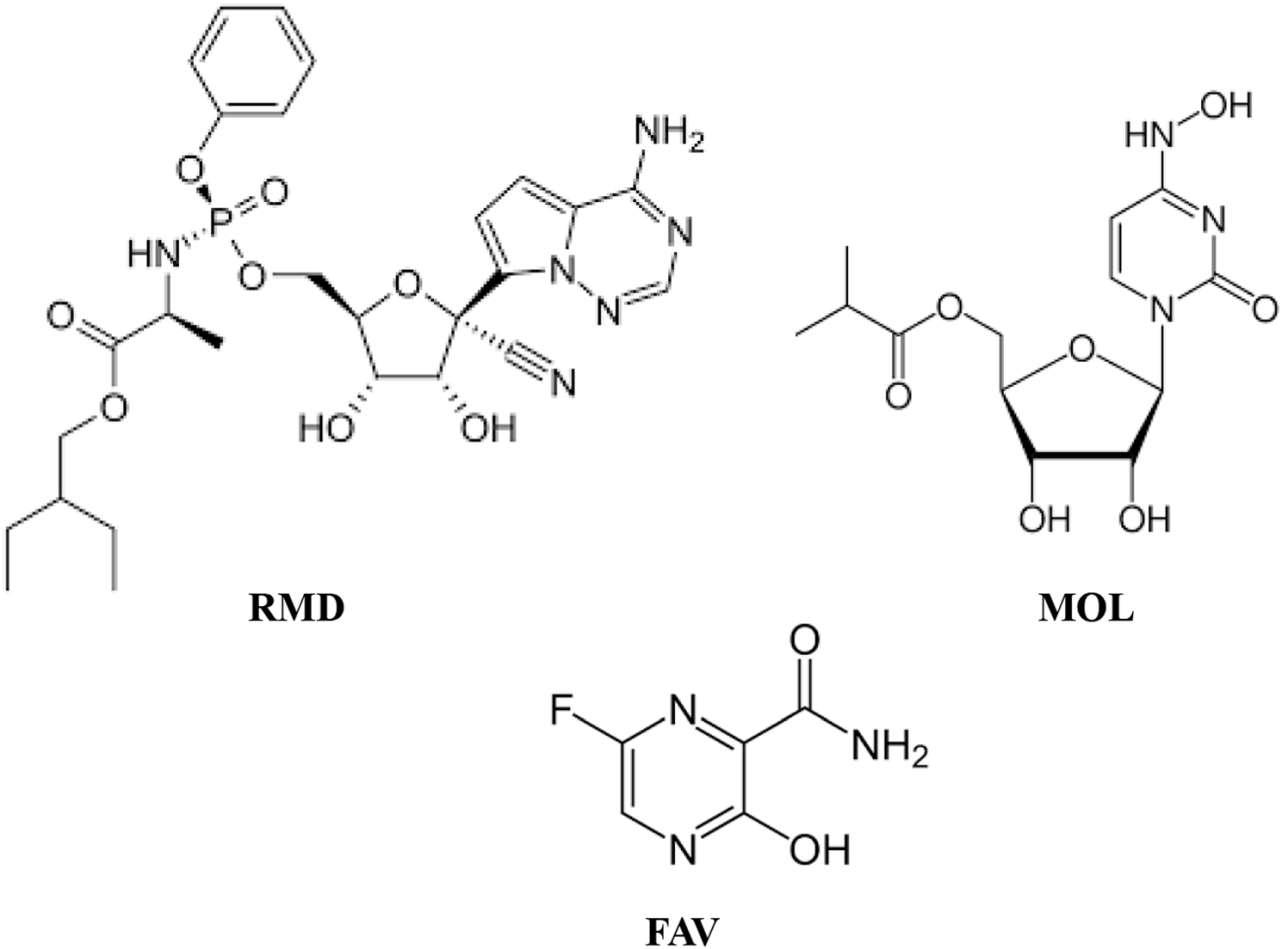
Chemical structures of Remdesivir (RMD), Molnupiravir (MOL), and Favipiravir (FAV)

Remdesivir (RMD) is 2-ethylbutyl (2 S)-2-[[[(2R,3 S,4R,5R)-5-(4-aminopyrrolo[2,1-f] [1, 2, 4] triazin-7-yl)-5-cyano-3,4-dihydroxyoxolan-2-yl] methoxy-phenoxyphosphoryl]amino]propanoate Fig. 1. It was previously produced by Gilead Science for treatment of Ebola viral infection [4]. The Covid-19 pandemic encouraged repurposing of the drug for treatment of Covid-19 infections after being subjected to additional clinical studies and it became the first FDA approved anticovid-19 drug [5, 6]. It is an adenosine triphosphate derivative prodrug. It works by inhibition of the viral RNA polymerase enzyme thus preventing the corona viral replication [7].

Favipiravir (FAV) is 6-fluoro-3-hydroxypyrazine-2-carboxamide, Fig. 1. This is a prodrug that was firstly developed in Japan by Toyama Chemical Company for treatment of influenza viral infection. After that, during Covid-19 pandemic, FAV was repurposed for treatment of Corona viral infection after emphasizing its efficiency in acceleration of viral clearance from infected body. After its in-vivo activation by phosphorylation, FAV acts as a viral RNA polymerase inhibitor [8–11].

Molnupiravir (MOL) is [(2R,3 S,4R,5R)-3,4-dihydroxy-5-[(4Z)-4-(hydroxyimino)-2-oxo-1,2,3,4-tetrahydropyrimidin-1-yl]oxolan-2-yl]methyl 2-methylpropanoate, Fig. 1. It is a prodrug that is metabolized into a ribonucleoside analog which is then converted to a phosphorylated derivative which induces mutations in the viral RNA replication [12]. MOL was originally produced to treat influenza infection [13]. In 2021, FDA issued an emergency use authorization (EUA) to MOL for treatment of mild to moderate COVID-19 infected patients who are not at high risk to develop severe illness [14, 15].

Nowadays, developing novel validated analytical methodologies with consideration of many diverse complementary criteria such as: cost-effectiveness, eco-compatibility, analytical efficiency, ease of application and good practicality, is regarded as the mainstay of quality control units. So far, the progressive environmental pollution worldwide and its resulted massive climate changes, conduce to regarding the pursuit of the principles of sustainable analytical chemistry as a crucial and mandatory task and no longer a luxurious option. The aforementioned facts have prompted us to develop two simple, sustainable, economic and practical HPTLC methods for the effective determination of RMD, FAV and MOL in bulk form and in pharmaceutical formulations. The herein proposed methods represent the first concurrent HPTLC determination of the three antiviral agents.

Upon surveying the literature, we have focused on the reported concomitant determinations of the three antivirals under study or at least two of them. Only a single recent report has tackled the simultaneous trace analysis of the three cited drugs in presence of the active metabolites of RMD and MOL in environmental aquatic samples (wastewater) using HPLC- HRMS (high resolution mass spectrometry) technique [16].

The concurrent estimation of RMD and MOL, was recently reported using UPLC [17] and HPLC [18] with PDA (photodiode array) detection in spiked human plasma with other co-administered drugs. Regarding the simultaneous determination of MOL and FAV, several reports were published employing different analytical techniques such as HPTLC [19] capillary electrophoresis and derivative ratio spectrophotometry [20], HPLC-PDA [21], LC-MS/MS [22], electrochemical techniques [23], synchronous spectrofluorimetry [24], micellar HPLC and mathematically assisted UV spectroscopy [25]. Concerning the concomitant quantification of RMD and FAV, a review article was published discussing the different reported analytical methods for their simultaneous determination [26]. Moreover, different reports using various techniques for concurrent determination of FAV and RMD were found including UPLC [27–30], HPLC [31], HPTLC [32], synchronous spectrofluorimetry [33, 34] and different spectrophotometric methodologies [35–37].

In the sake of providing tangible evidence on the designed methods’ outstanding sustainability and practicality, multiple complementary metrics were applied to assess the methods’ greenness, blueness and whiteness as well as to prove their superiority against the only reported HPLC-HRMS technique. These tools are the Analytical Eco-Scale [38], the recently launched MoGAPI (September 2024) [39] and AGREE [40] metrics to appraise the methods’ greenness. The recent BAGI [2] metric and the RGB12 algorithm [1] were employed to judge their applicability (blueness) and sustainability (whiteness), respectively. This thorough integrative trichromatic evaluation provided a pragmatic proof on the proposed methods’ excellent sustainability.

Lately, HPTLC technique is the advanced alternative form of planar chromatography. This technique is flexible, versatile and has a promising potential for automation, hyphenation and upgrading. In spite of the fact that HPLC is still considered as the gold standard of analytical techniques due to its widespread availability and detector versatility, currently, HPTLC excels in attracting the attention of analysts being a more economic, eco-compatible, simpler and faster alternative to HPLC specially the costly and complicated HPLC-MS. HPTLC affords a lot of notable merits versus HPLC such as: faster analysis, easier operation, lower volume of sample and waste output, less maintenance cost and concomitant analysis of several samples on the same plate in a parallel rather than sequential mode allowing greater analytical throughput. Moreover, HPTLC is lately successfully subjected to novel hyphenation with advanced detectors such as: Fourier transform infrared (FTIR), mass spectrometer (MS) and laser spectroscopy which facilitates its adoption in huge variety of applications in many fields of sciences. Indeed, HPLTC still has a great potential for further development to cope with more advanced fields of applications [41, 42].

In the present study, these three anti-Covid-19 agents are specifically selected as they are the only FDA approved single drug treatments of Covid19. Indeed, they are not co-administered together, although, a recent report confirmed the effectiveness of an antiviral combination of the two oral anti-Covid FAV and MOL as it aided in an efficacious 60% eradication of SARS-CoV2 in hamsters and the report recommended the design of a human clinical study for this combination treatment [43]. On the other hand, most of the drug manufacturing companies afford the single drug formulations of these three agents. We suggest these companies will greatly profit from the newly designed HPTLC methods which afford cost effective, sustainable and practical multi-component analysis tool. The proposed methods will facilitate the analysis of the 3 drugs in bulk form or samples of the their corresponding three produced formulations concurrently using the same TLC plate and same mobile phase in a parallel manner in the same chamber in quality control labs. Hence, the designed methods are suggested to be feasible and favorable for implementation in quality control laboratories, particularly, those located in drug manufacturing companies that concomitantly manufacture the single drug formulations of the three antiviral agents under study. They will save money, effort and energy without harmful effects on the environment and the operator’s health and safety.

## Experimental

### Instrumentation and software

Accurate weights of the analytes were performedusing a 4-digits Viber electronic balance. The stock and dosage form solutions were dissolved and extracted using a sonicator water-bath. Bands of the samples were applied on the TLC plates using CAMAG Hamilton microliter syringe (100 µL capacity) under nitrogen stream supported by a CAMAG Linomat IV sample applicator (Switzerland). In this work, the application was carried out on precoated TLC silica gel aluminum plates 60 F_254_ (20 × 10 cm, 200 μm thickness, Merck, Darmstadt, Germany) for HPTLC-NP (method I) and TLC silica gel plates 60 RP-18 F_254_ (20 × 10 cm, 200 μm thickness, Merck, Darmstadt, Germany) for HPTLC-RP (method II). A CAMAG twin trough glass chamber (20 × 20 cm) was saturated with the mobile phases for 15 and 35 min at room temperature for methods I & II, respectively, before the plate development. CAMAG TLC scanner III controlled with WinCATS software (V 3.15 CAMAG) was utilized for densitometric scanning. The greenness assessment was calculated and sketched using freely available software of analytical GREEnness calculator (AGREE) (downloadable from https://mostwiedzy.pl/AGREE.) and MoGAPI (https://fotouhmansour.github.io/MoGAPI/) software. To evaluate method practicality and applicability, the online BAGI software (https://bagi-index.anvil.app/) was used to calculate BAGI score. Additionally, evaluation of the methods’ whiteness was performed using freely available Excel spreadsheet available at (https://view.officeapps.live.com/op/view.aspx?src=https%3A%2F%2Fars.els-cdn.com%2Fcontent%2Fimage%2F1-s2.0-S0165993621000455-mmc2.xlsx&wdOrigin=BROWSELINK).

### Materials and reagents

Pure powder of RMD, FAV and MOL (certified potencies are 99.7%, 99.5% and 99.2%, respectively) were kindly donated by EVA Pharma Co., Egypt. HPLC grade absolute ethanol (Fisher Scientific, Loughborough, UK) and ethyl acetate (El-Nasr Pharmaceutical Chemicals Co., Qaliubiya, Egypt) were used in this investigation. The pharmaceutical preparations used in the current work were Remdesivir^®^ concentrate solution for IV infusion containing 100 mg RMD per 20 mL vial (B.N. 2105598 A, Eva pharma, Giza, Egypt), Avipiravir^®^ film coated tablets (B.N. 2202530R, Eva pharma, Giza, Egypt) containing 200 mg FAV per tablet B.N. 2202530R and Molnupiravir- EVA Pharma^®^ hard capsules (B.N. 2405329, Eva pharma, Giza, Egypt) containing 200 mg MOL per capsule. Inactive ingredients used for preparation of placebo solutions are: Betadex sulfobutyl ether sodium, sodium hydroxide and hydrochloric acid dissolved in water for injection. In addition to, low substituted hydroxypropyl cellulose, povidone K30, colloidal silicon dioxide, crospovidon, sodium stearyl fumarate, hydroxypropyl methyl cellulose, titanium dioxide, talc, microcrystalline cellulose, magnesium stearate, hypromellose and titanium dioxide (all inactive ingredients are kindly donated from Eva pharma drug manufacturing company, Giza, Egypt).

### General procedures

#### Chromatographic conditions

An accurate volume of 5 µL of each sample was applied by a CAMAG microliter syringe. The settings of the applicator were adjusted at 7 mm from the margin, 10 mm from the bottom edge of the plate, 5 mm bandwidth and 4 mm inter-spaces between successive bands. For method I, 15 mL of a mobile phase system composed of ethyl acetate, ethanol and water (94: 4: 2.5) was placed in the glass chamber. For method II, 25 mL of a mobile phase system consisting of ethanol and water (60: 40) was used.

The plates were placed in the glass chamber after its saturation with the mobile phases for 15 and 35 min at ambient temperature (25 ± 2 °C) for method I and II, respectively. Then, the plates were ascendingly developed over nearly 9.8 cm. The developed plates were left to air-dry and finally densitometric scanning was done at 244 nm for RMD and MOL and at 325 nm for FAV.

#### Construction of the calibration graphs

Separate stock solutions were prepared in absolute ethanol of concentration 1000 µg/mL of RMD, FAV and MOL. Precisely measured volumes of stock solutions corresponding to 30–800 µg of RMD and 50–2000 µg of FAV and MOL were transferred into 5 mL volumetric flasks. Adjustment of volumes to 2.5 mL was done using absolute ethanol and then dilution to the mark with distilled water was performed. From each working solution, 5 µL were spotted on the TLC plates to get final concentration ranges of 30–800 ng/band for RMD and of 50–2000 ng/ band for both FAV and MOL. The construction of calibration graphs was performed correlating the average peak areas (average of 3 spotted samples for each concentration) of the cited drugs to their relevant concentrations and the linear regression equations were calculated.

### Assay of pharmaceutical preparations

#### Assay of Remdesivir concentrate solution for IV infusion

In a 10 mL volumetric flask, accurate volume of 2 mL of Remdesivir^®^ vial concentrate solution (100 mg RMD per 20 mL vial), equivalent to 10 mg RMD, was diluted to 5 mL with distilled water then made to mark with absolute ethanol to obtain a final concentration of 1000 µg/mL RMD (stock sample solution). A placebo solution was supplied by EVA Pharma drug manufacturing company, containing the inactive ingredients (excipients mentioned in the pamphlet of the pharmaceutical formulation). The inactive ingredients are: Betadex sulfobutyl ether sodium, sodium hydroxide and hydrochloric acid dissolved in water for injection.

#### Assay of Avipiravir® film coated tablets

Ten tablets were accurately weighed and finely powdered. Accurate weight of the powdered tablets corresponding to 50 mg FAV was extracted into 40 mL absolute ethanol with the aid of sonication for 20 min then filtered into separate 50 mL-volumetric flask. Washing of the residue was performed with volumes of ethanol and added to the filtrate till reaching the volumetric flask mark to obtain final concentration 1000 µg/mL FAV (stock sample solution). A placebo solution was prepared containing the inactive ingredients (excipients mentioned in the pamphlet of the pharmaceutical formulation and supplied by EVA Pharma drug manufacturing company) sonnicated with absolute ethanol then filtered. The inactive ingredients are: low substituted hydroxypropyl cellulose, povidone K30, colloidal silicon dioxide, crospovidon, sodium stearyl fumarate, hydroxypropyl methyl cellulose, titanium dioxide and talc.

#### Assay of Molnupiravir- EVA Pharma® hard capsules

The content of ten capsules were accurately weighed and thoroughly mixed. Accurate weighed part of the powder analogous to 50 mg MOL was extracted into 40 mL absolute ethanol, sonicated for 20 min and then filtered into separate 50 mL-volumetric flask. The residue was washed with portions of ethanol and washings were added to the filtrate till reaching the volumetric flask mark to obtain final concentration 1000 µg/mL MOL (stock sample solution). A placebo solution was prepared containing the inactive ingredients (excipients mentioned in the pamphlet of the pharmaceutical formulation and supplied by EVA Pharma drug manufacturing company) sonnicated with absolute ethanol then filtered. The inactive ingredients are: microcrystalline cellulose, magnesium stearate, hypromellose and titanium dioxide.

Exactly measured volumes of the stock sample solutions were delivered to 5 mL volumetric flasks and made up to volume with 50% aqueous ethanol to reach their specified concentration ranges. The working sample solutions were then treated as under “General Procedures”. Similarly, the three placebo solutions of the analyzed formulations were treated and analyzed. For external standard method, the percentage recoveries were calculated using analogously prepared standard solutions. Concerning standard addition assay, it was carried out by accurate addition of different portions of standard solutions of the analytes to its sample solutions to obtain total concentrations within their specified ranges then analyzed as formerly mentioned. Calculation of recovered concentrations was performed by comparing the analyte response with the rise in response attained by the standard solution added.

## Results and discussion

### Optimization of chromatographic conditions

Optimization of chromatographic conditions for each HPTLC method employing different stationary phases was carried out regarding the best mobile phase components, the parameters of application of bands and the selected detection wavelengths to attain best resolution of the analyzed drugs with symmetric and sharp peaks possessing appropriate R_f_ values.

**Fig. 2:**
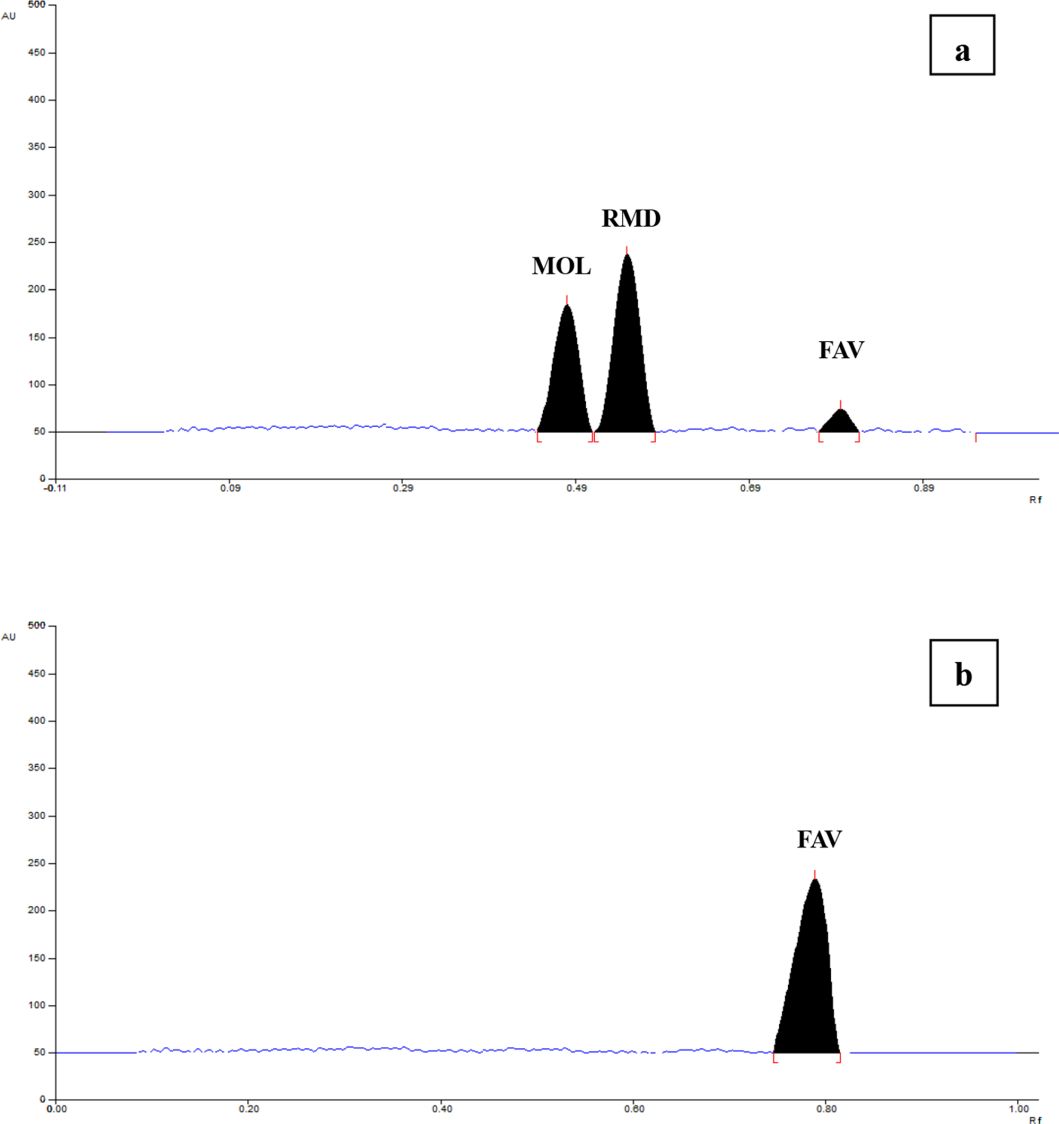
Densitogram of 600 ng/band MOL, 600 ng/band RMD and 1000 ng/band FAV at 244 nm (**a**) and at 325 nm (**b**) using HPTLC-NP method

Concerning the mobile phase components, for the sake of designing sustainable methodologies, we have tried different proportions of ecologically benign components. Hazardous solvents such as toluene, chloroform and benzene etc. are avoided because of their environmental and health hazards. The HPTLC-NP method employed the stationary phase consisted of Merck TLC plates precoated with 60 F _254_ silica gel on aluminium sheet for normal-phase separations which required predominantly non-aqueous mobile phases. For this method, we started with trying various proportions of bio-based solvents such as ethyl acetate and ethanol. In this case, unfortunately, the developed drugs’ peaks suffered from broadness in shape and poor resolution between MOL and RMD peaks, also FAV peak travelled quickly to the solvent front line. Upon replacing ethanol with the less green methanol, no improvement in peak shape occurred. Depending on the fact that, the three drugs are sparingly soluble in water, therefore the addition of a small portion of distilled water to the mobile phase mixture provided reasonable retention of FAV on the TLC plate and improves the resolution between RMD and MOL. Moreover, water added to the mobile phase afforded symmetrical shape for the three developed drugs’ peaks. The best selected solvent system consisted of ethyl acetate: ethanol: water (9.4: 0.4: 0.25, v/v/v, respectively). Ratio of water is very critical (2.5% of mobile phase volume), as greater ratio is immiscible with the added portions of ethyl acetate and ethanol, while lower ratio resulted into poor resolution of MOL and RMD peaks. Consequently, well-resolved peaks of MOL, RMD, and FAV were detected at R_f_ values of 0.47 ± 0.02, 0.54 ± 0.02 and 0.80 ± 0.02, respectively (Fig. 2).

**Fig. 3:**
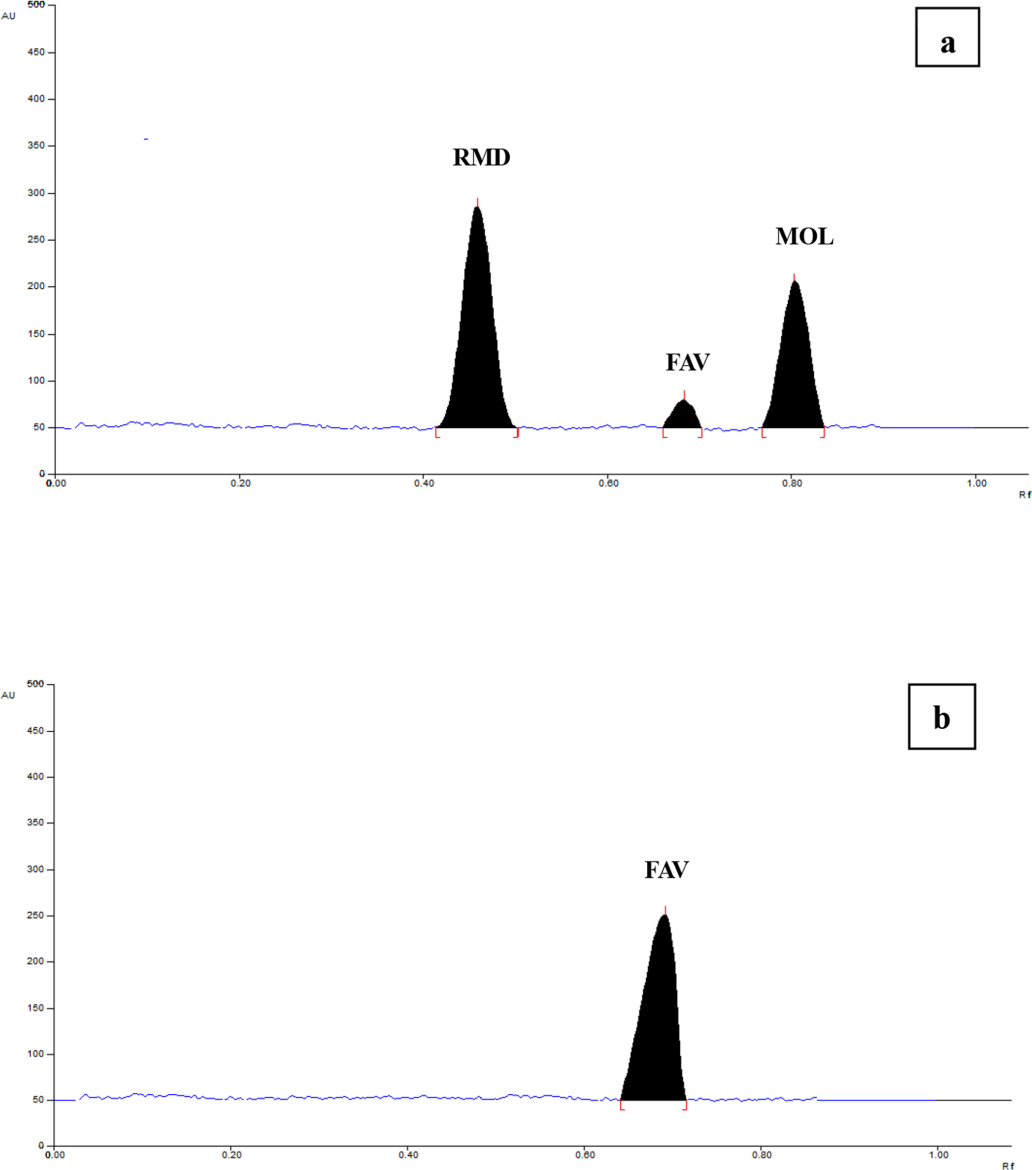
Densitogram of 600 ng/band RMD, 1000 ng/band FAV and 600 ng/band MOL at 244 nm (**a**) and at 325 nm (**b**) using HPTLC-RP method

For HPTLC-RP method employed TLC plates consisted of reversed-phase layers prepared from dichloroalkylmethylsilanes with octadecyl- (RP-18) alkyl groups where the organosilane reagent is bonded to the surface by a combination of one or two bonds. For this method more polar ecologically benign solvents were favorable. We attempted different proportions of the greenest solvents, ethanol and water. The optimum ratio selected was ethanol: water (6:4, v/v respectively). Replacing ethanol with the less green acetone resulted into FAV peak broadening with poor resolution between FAV and MOL. Lower ratio of ethanol caused poor migration of RMD band from the baseline. Lower ratio of water resulted in poor resolution of FAV and MOL peaks. Greater ratio of water (> 40%) is not preferred as it resulted into delay of mobile phase migration because the hydrophobic repulsive forces will be much higher than the capillary forces migrating the solvent through the layer. Additionally, high ratio of water may cause swelling and peeling of the precoated layers from the support [44]. Under the selected mobile phase ratio, RMD, FAV and MOL migrated with symmetric and well resolved peaks detected at R_f_ values of 0.46 ± 0.02, 0.69 ± 0.02 and 0.81 ± 0.02 respectively (Fig. 3).

Regarding the volume of mobile phase and saturation time, they differ between both methods as they greatly depend on the volatility of mobile phase components. Solvent volatility increases as its boiling point (B.p.) decreases and consequently its vapor pressure (Vap. P.) increases; this will lead to faster achievement of full chamber saturation by employing lower volume of mobile phase. Concerning the HPTLC-NP method, 94% of mobile phase volume consists of ethyl acetate (B.p. = 77ºC and Vap. P. = 9.7 KPa), 4% ethanol (B.p. = 78.24ºC and Vap. P. = 5.83 KPa) and only 2.5% water (B.p. = 100ºC and Vap. P. = 2.31 KPa). In contrast, 60% of the HPTLC-RP mobile phase volume consists of ethanol and 40% of the volume is water. The aforementioned facts explain the higher volatility of the HPTLC-NP method’s mobile phase thus only 15 mL are needed to achieve full chamber saturation in 15 min. While a greater volume is needed (25 mL) from the less volatile HPTLC-RP method’s mobile phase and longer time (35 min) is needed to reach full chamber saturation [45].

Furthermore, to ensure the good performance of the developed HPTLC methods, the system suitability parameters were calculated, and they were found convenient in compliance with the reported reference values [46] (Table S1 in supplementary file 1).

Considering the choice of band width and the space between bands, different sizes of bands were tried (4, 5, and 8 mm width); similarly, interspaces of 4, 5, and 8 mm were checked to achieve a good concurrence of well-symmetrically resolved peaks and the greatest number of bands (samples) reachable to apply on a single plate. The best bandwidth selected was 5 mm with leaving 4 mm as inter-band space. These dimensions granted the spotting of up to 20 samples (20 bands) per plate (10 × 20 cm) with affording reasonable symmetry and good sharpness of the resolved peaks.

The selection of detection wavelength depended on the maximum absorption of each of the analyzed drugs to afford the best sensitivity of their determination. The ideal scanning wavelengths were 244 nm for detection and quantification of RMD and MOL (isosbestic point), while 325 nm was chosen for FAV (maximum absorption wavelength). Figure S1 (in supplementary file 1) showcases the UV spectra of the three analyzed drugs.

### Validation of the proposed methods

The process of validating the proposed HPTLC methods was conducted as stated by the principles set out by the newly endorsed ICH [Q2(R2)] on validation of analytical procedures [47].

#### Linearity and concentration ranges

Linearity of each of the cited drugs was inspected under the pre-mentioned operating conditions. Then, the calibration curves were created by using the least square method. It was observed that the measured peak areas of the studied drugs in both methods were perfectly proportional to their relevant concentrations over concentration ranges of 30–800 ng/spot of RMD and of 50–2000 ng/spot of both FAV and MOL. The indication of excellent linearities of the proposed methods is high correlation coefficient values (not less than 0.99988) together with low RSD% value of the slopes (not more than 0.698). The linearity data and statistical parameters are shown in Table 1.

**Table 1:** Analytical parameters for the determination of RMD, FAV and MOL using the proposed HPTLC methods

Parameter	NP-HPTLC			RP-HPTLC		
	RMD	FAV	MOL	RMD	FAV	MOL
Detection wavelength (nm)	244	325	244	244	325	244
**Range** **ng/Band** (µg/mL)	**30–800** (6-160)	**50-2000** (10–400)	**50-2000** (10–400)	**30–800** (6-160)	**50-2000** (10–400)	**50-2000** (10–400)
**Intercept (a)**	10.530	50.439	10.104	6.622	-8.639	-11.214
**Slope (b)**	8.157	8.020	5.290	11.662	7.040	7.082
**Correlation coefficient (r)**	0.99988	0.99995	0.99999	0.99994	0.99999	0.99998
**S**_**a**_	23.710	37.793	10.771	24.374	12.314	17.462
**S**_**b**_	0.057	0.037	0.011	0.057	0.012	0.018
**RSD% of the slope**	0.699	0.461	0.208	0.489	0.170	0.254
**S**_**y/x**_	41.635	67.657	19.871	42.299	22.044	32.213
**Detection Limit (DL)** **ng/Band** (µg/mL)	**9.592** (1.918)	**15.551** (3.110)	**6.719** (1.344)	**6.897** (1.379)	**5.772** (1.154)	**8.137** (1.627)
**Quantitation Limit (QL)** **ng/Band** (µg/mL)	**29.067** (5.813)	**47.123** (9.425)	**20.361** (4.072)	**20.900** (4.180)	**17.491** (3.498)	**24.657** (4.931)

#### Limit of detection and quantification

Detection and quantitation limits (DL and QL) were estimated in accordance with the formula, DL = 3.3 σ/S, QL = 10 σ/S (ICH); where σ signifies the standard deviation of y-intercepts of the regression line and S represents the slope of the calibration curve. The values of DL and QL of different analytes were presented in Table 1.

#### Accuracy and precision

The repeatability (within-day precision) and intermediate precision (between-day precision) of sample application and peak area measurement were assessed by triplicate spotting of three distinct concentrations at low, medium and high levels within the linearity range of each analyte on a single day (for intra-day precision and accuracy) and on three consecutive days (for inter-day precision and accuracy). Table 2 sums up the obtained analytical results where the percentage relative standard deviation (RSD %) and percentage relative error (E_r_ %) were found to be less than 1.5% (within limits; ≤ 2%) proving the excellent precision and accuracy of the proposed methods.

**Table 2:** **Precision and accuracy for the analysis of RMD**,** FAV and MOL in their bulk form using the proposed methods.**

Method	Drug	Conc. (ng/Band)	Type of analysis	Mean^a^ % recovery	SD^a^	RSD^a^%	E_*r*_%
**HPTLC-NP**	**RMD**	200	**Intra-day**	100.15	0.08	0.08	0.15
		400		100.04	0.10	0.10	0.04
		600		100.10	0.03	0.03	0.10
		200	**Inter-day**	100.51	0.18	0.18	0.51
		400		100.35	0.23	0.23	0.35
		600		100.20	0.07	0.07	0.20
	**FAV**	400	**Intra-day**	99.91	0.12	0.12	-0.09
		800		100.02	0.14	0.14	0.02
		1400		99.95	0.09	0.09	-0.05
		400	**Inter-day**	100.13	0.46	0.46	0.13
		800		100.08	0.34	0.34	0.08
		1400		99.86	0.52	0.52	-0.14
	**MOL**	400	**Intra-day**	99.98	0.19	0.19	-0.02
		800		99.99	0.12	0.12	-0.01
		1400		100.05	0.10	0.10	0.05
		400	**Inter-day**	100.07	0.20	0.20	0.07
		800		100.17	0.41	0.41	0.17
		1400		100.18	0.15	0.15	0.18
**HPTLC-RP**	**RMD**	200	**Intra-day**	99.62	0.32	0.32	-0.38
		400		100.19	0.09	0.09	0.19
		600		100.10	0.06	0.06	0.10
		200	**Inter-day**	99.82	0.59	0.59	-0.18
		400		100.35	0.55	0.55	0.35
		600		100.77	0.51	0.51	0.77
	**FAV**	400	**Intra-day**	100.04	0.28	0.28	0.04
		800		100.14	0.15	0.15	0.14
		1400		100.08	0.10	0.10	0.08
		400	**Inter-day**	100.10	0.79	0.79	0.10
		800		99.99	0.29	0.29	-0.01
		1400		100.16	0.50	0.50	0.16
	**MOL**	400	**Intra-day**	100.62	0.29	0.29	0.62
		800		99.87	0.16	0.16	-0.13
		1400		100.09	0.03	0.03	0.09
		400	**Inter-day**	101.41	0.40	0.39	1.41
		800		100.08	0.43	0.43	0.08
		1400		100.57	0.38	0.38	0.57

^a^ Mean, RSD% and SD of three determinations

#### Selectivity

Methods’ selectivity was tested by the preparation of synthetic mixtures at various ratios of pure RMD, FAV and MOL, followed by their analysis using the proposed methods. Satisfactory results of recovered concentrations, RSD% and Er% were obtained and depicted in Table S2 (in supplementary file 1). The results were found within limits (≤ 2%) proving the successful selectivity of the established HPTLC techniques in resolving and quantifying cited drugs in mixtures of variant concentration ratios.

#### Robustness

Robustness of the studied methods was appraised by making deliberate intended variations in parameters such as the working wavelength (± 2 nm), mobile phase composition and volume, time from chromatography to scan and duration of chamber saturation with the mobile phase then recording the measured peak areas and R_f_ values of the cited compounds. Good robustness of the suggested methods was guaranteed through satisfactory RSD% values of peak areas along with small changes in R_f_ values, signifying the reliability of the proposed methods during the routine work. Tables S3 and S4 (in supplementary file 1) summarize the robustness results of the proposed methods.

#### Stability of solutions

The stability of the stock solutions was assessed by repeating their analysis every day while storage in the refrigerator for the next day. It was investigated that the stock solutions were stable for at least 3 days. Also, the stability of working solutions at room temperature was checked along 6 h (on the same day) where the changes observed are within the relative standard deviation (RSD %) limit (≤ 2%), approving the stability of the working solutions.

### Application of the proposed methods on analysis of the different pharmaceutical preparations

Upon the application of the suggested methods, excellent quantification of RMD, FAV and MOL was observed while analyzing their pharmaceutical formulations without any interfering peaks from the excipients (Figures S2, S3 and S4 in supplementary file 1). The conducted analysis using external standard and standard addition methods yielded good recoveries not less than 99.18% with acceptable accuracy and precision (Tables S5 and S6 in supplementary file 1). The low RSD% values (less than 1.42%) confirm the appropriateness of these methods for the routine analysis of the studied drugs in their pharmaceutical dosage forms.

Specificity is defined as the ability to access unequivocally the analyte in the presence of components that may be expected to be present, such as impurities, degradation products and matrix components [47]. Study of the specificity of the proposed approaches was authorized by the successful separation of analyzed drugs. Moreover, ascertaining the specificity through the peak purity profiling in both methods was carried out. This was done across each drug peak through recording the UV absorption spectrum at several points by the TLC scanner and calculating two correlation coefficients (r_s, m_ at peak start and peak maximum and r_e, m_ at peak end and peak maximum) by the software [48]. After that, the software decides if the selected peak is pure or not. Drug spots from both standard and pharmaceutical formulations were tested for purity. Figures S5 a and b (in supplementary file 1) depict the peak purity of the drugs’ spots. The spectra of standard and sample solutions were found to be superimposed with high correlation coefficients not less than 0.9993. Moreover, the absence of any eluted peaks in the analyzed placebo solutions (formulations’ matrices) at the specific R_f_ values of the cited drugs indicates that there is no interference caused by commonly used excipients in different analyzed dosage forms.

Furthermore, by the one-way analysis of variance test (Single factor ANOVA), the results of the adopted methods and the two reference HPTLC methods [19, 32] were statistically compared. The ANOVA test was performed for comparison of data obtained from more than two methods [49]. It was depicted that the calculated F-values were lower than the tabulated values, verifying no discernible differences between different adopted and reference approaches (Table S5 in supplementary file 1). The adopted and reference procedures are successfully appropriate to the quantification of examined drugs in their pharmaceutical formulations with optimum and analogous analytical performance.

### Trichromatic integrative assessment and comparison: green (Ecological Safety), Blue (Applicability) and White (Sustainability)

The pursuit of sustainability in analytical methodologies requires the integrative implementation of eco-compatible reagents, accomplishment of excellent analytical performance and achievement of high practicality and simple applicability of newly developed method. In the current study, we have conducted a thorough trichromatic (green, blue and white) evaluation and comparison of sustainability by integrative employment of multi-complementary metrics to encompass the diverse pillars of sustainability [50]. To claim that an analytical method is sustainable, this requires providing a tangible evidence and pragmatic proof through excellent synergistic results of several complementary sustainability metrics regarding this method [51–53]. Consequently, this helps in providing a reasonable judgment and considered decision about the degree of sustainability of the method. This also allows a clear discrimination between different analytical procedures in terms of their adherence to the concept of sustainability. In the current study, we evaluate the 2 designed HPTLC methods along with the only recently reported HPLC-HRMS method [16] which simultaneously analyzes the 3 drugs under study.

#### Greenness appraisal and comparison

Recently, it was reported that the most frequently cited green metrics, for evaluation of the environmental impact (greenness) of new and current analytical procedures, are the analytical eco-scale [38] and the conventional GAPI metrics [39].

The Analytical Eco-Scale [38] assesses many aspects of the analytical process including the type and volume of solvents and reagents employed in the method and their hazards, the consumed energy, and the volume of waste output. This tool assigns penalty points for each unfulfilled green character. A score of 100 is regarded as the fully green method’s score. Total penalty points of each method are then deducted from 100. The higher the final score the greener is the analytical procedure ranging from excellent (score ≥ 75) to acceptable (score ≥ 50) to inadequate green (score < 50). Upon applying this metric on the methods under study, the proposed HPTLC-RP obtained the highest score (92, i.e. lowest penalty points) followed by the HPTLC-NP method (90) then the lowest score (76, i.e. highest penalty points) was obtained by the reported HPLC-HRMS method. These results assert the excellent green character of the proposed HPTLC methods which excel the reported method [Table 3 and S7 (in supplementary file 1)].

**Table 3:**
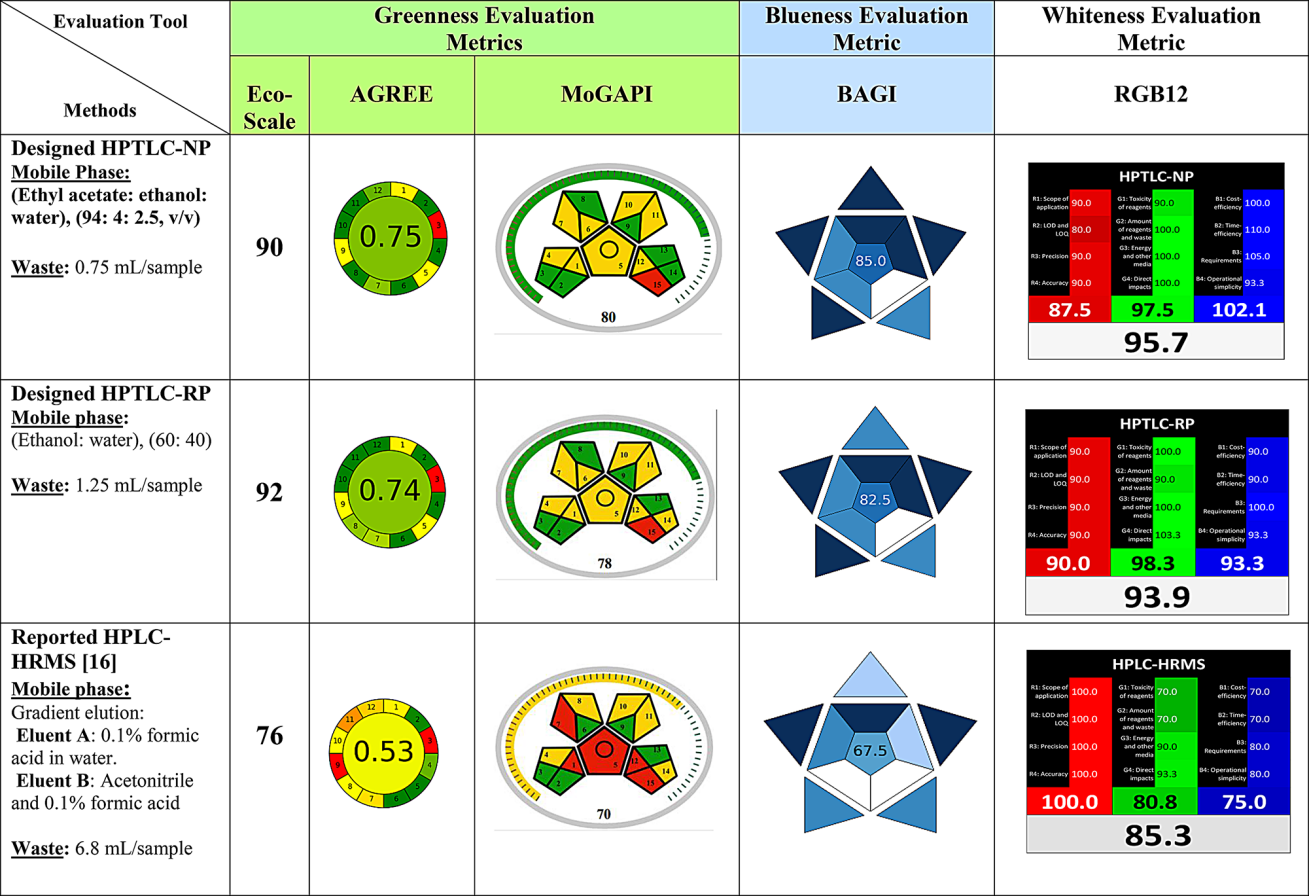
Integrative Multi-metric evaluation of sustainability of designed HPTLC methods and comparison with reported method [16]

The conventional GAPI metric is now modified to more insightful and informative measures such as the recently launched Modified GAPI (MoGAPI) tool in September 2024 [39]. This newly published metric resembles the conventional GAPI in the evaluation of the entire analytical methodology through individually appraising 15 different specific aspects starting from sampling and going through the method’s type (qualitative or quantitative), sample preparation, reagents and solvents volume and their hazards and energy consumption until reaching the volume of produced waste and any possible treatment of this waste. The result is then showcased in the form of pictogram consisted of 15 colored zones relevant to the 15 appraised ecological parameters. The novel MoGAPI not only allocates the distinctive green, yellow and red colors to the different 15 zones (decoding low, medium and high ecological hazards) but also assigns a final holistic percentage score as well as a colored scale surrounding the 5 pentagrams of GAPI. This represents an overall assessment of the method’s greenness facilitating both quantitative and qualitative comparison between different methods. It also excels over the traditional GAPI in being simply and expeditiously calculated using a freely available software. After applying this metric on the three methods, the HPTLC-NP method has acquired a total score of 80 (the highest green method) with a green-colored total scale followed by a score of 78 assigned to the HPTLC-RP method with also a green-colored overall scale. Akin to previous Eco-Scale results the reported HPLC-HRMS has obtained the lowest MoGAPI score of 70 with a yellow-colored overall scale around the pentagrams (the lowest green method) (Table 3) (all input data of MoGAPI metric are represented in supplementary file 2).

The AGREE tool [40] is a more intuitive tool evaluating the 12 principles of GAC representing most of its stipulations including the previously assessed parameters by The Analytical Eco-Scale and MoGAPI tools with consideration of some previously disregarded parameters such as the analytical throughput of the method. AGREE provides both visual inspection and quantitative declaration of the degree of greenness of the evaluated procedure. The result is generated in the form of colored circular pictogram with a final central score (0–1) summarizing the results of the surrounded 12 segments relevant to the 12 appraised parameters of the method. The more the central score is near to 1 the greener is the method. The 12 segments are also colored from dark green to red identifying the parameters needing improvement in the method to achieve more adherence to the GAC concept. Upon application of this tool on the three compared methods, likewise the MoGAPI results, the HPTLC-NP has got the highest score of 0.75 followed by a comparable score of 0.74 obtained by the HPTLC-RP method emphasizing the eminent eco-friendliness of the designed methods. Finally, the lowest score of 0.53 was acquired by the published HPLC-HRMS method decoding the most ecologically harmful method (Table 3) (all input data of AGREE metric are represented in supplementary file 2).

The previous findings are attributed to the multistep character of HPLC-HRMS method involving solid phase extraction of samples before analysis, more energy is consumed by the device (LC-MS), greater volume of waste is generated with low analytical throughput (only 2 samples are analyzed per hour) compared to higher analytical throughput of both designed HPTLC methods [HPTLC-RP (8 samples per hour) and HPTLC-NP (39 samples per hour) in addition to lower energy consumption and less volume of waste. Moreover, the operator is subjected to more threats in the HPLC-HRMS method due to employment of acetonitrile, methanol and formic acid in contrast to the proposed HPTLC methods where the HPTLC-RP is devoid from any threats due to the use of totally green solvents and mobile phase components (ethanol and water) and the HPTLC-NP subjects the operator to only one threat which is the high flammability of ethyl acetate.

Indeed, the previously applied tools afford a multidimensional assessment and comparison of greenness from both quantitative and qualitative attributes. Nevertheless, they still lack the inspection of other methods’ criteria such as the economic expediency, the degree of analytical performance as well as the measure of practicality and applicability of the methods.

#### Evaluation of practicality using BAGI metric

The novel BAGI metric (Blue Applicability Grade Index) [2] effectively evaluates the analytical method’s practicality and applicability. BAGI is complementary to the applied green appraisal metrics. This tool symbolizes the method’s functionality by means of a colored astral pictogram and overall score in its center ranging from 25 to100. It is easily calculated using a freely available web application that expedites the discrimination between different analytical methods’ functionality at a first glance. BAGI assesses 10 characters of the procedure related to the sample preparation and determination steps encompassing the analysis type, the extent of simultaneous preparation and determination of samples, samples volume, the availability of the employed reagents and solvents as well as the availability and degree of automation of the applied analytical technique. In the context of fit-for-purpose concept, the aforementioned criteria are crucial for a method to be applicable and greatly affect the selection of the analytical procedure by routine analysis laboratories and accreditation labs as well. Comparing the BAGI scores of the developed methods and the reported one, give valuable insights into the real-world practicality of the designed HPTLC methods versus the existing HPLC-HRMS procedure. The superiority of our methods in terms of practicality is well illustrated by the high GABI scores (85 for HPLC-NP and 82.5 for HPTLC-RP) versus the low score (70) obtained by the existing HPLC-HRMS procedure (Table 3) (all input data of BAGI tool are represented in supplementary file 2). These findings are mainly attributed to the widespread availability of the employed reagents in the proposed methods, more availability of the employed HPTLC technique than HPLC-MS, and high analytical throughput which accounts for faster analysis and consequently saves time, energy and money. These findings seem very attractive to approve the implementation of the proposed methods in quality control and accreditation labs.

Despite the effectiveness of BAGI in evaluating the functionality of proposed methods, it lacks providing a holistic outline of the entire methods’ sustainability. Accordingly, to fulfill a thorough evaluation of the multifaceted sustainability enclosing its 3 pillars: the greenness, analytical performance and practicality, we furthermore applied the RGB12 [1] tool to assess the methods’ whiteness.

#### Appraising and comparing the whiteness of the developed methods versus the reported one

White analytical chemistry (WAC) is complementary to GAC with enclosing three important pillars: the analytical merit, the eco-compatibility, as well as the applicability aspects of the analytical methodology expressed as the red-green-blue (RGB) algorithm [1]. In WAC, four main attributes are assessed in each pillar. The data are fed into an open source excel spread sheet and the evaluation results are automatically calculated and formatted in the form of simple tables which could be easily construed. The individual red, green and blue scores are added within each color category and then combined to provide a final score from 0 to 100. As the overall score is closer to 100, this means better alignment of the method with sustainability (whiteness) aspects. Table 3 and S8 (in supplementary file 1) illustrate the whiteness scores obtained by the proposed and reported methods. The developed HPTLC-NP and HPTLC-RP methods conform with the 12 sustainability characters and obtained high whiteness scores of 95.7 and 93.9, respectively. On the other hand, HPLC-HRMS acquired a lower score of 85.3 which is attributed to its poor functionality and practicality and its negative ecological impacts by subjecting the operator and environment to unavoidable threats.

It is worth mentioning that the developed HPTLC-NP excels the HPTLC-RP method albeit employing a little bit less green mobile phase. This is attributed to the higher practicality of the HPTLC-NP expressed by short analysis time and high analytical throughput. This is because of the high volatility of the applied mobile phase components (ethyl acetate: ethanol: water, 9.4:0.4:0.25; v/v) leading to shorter time needed for chamber saturation, faster development and faster dryness of plates before scanning. This is not effectively achieved in HPTLC-RP method, although its totally green mobile phase components (ethanol: water, 6:4; v/v) due to its low volatility as it comprises 40% water.

## Study limitations and future prospects

While the currently designed HPTLC methods demonstrate a superb performance, sustainability and practicality in analyzing the three cited drugs, there are still avenues for future improvement. Most notably, the current study lacks the implementation of analytical quality by design (AQbD) approach during the step of method development established on the fundamentals of quality risk management (QRM) and design of experiments (DoE) [53–56]. AQbD identifies and explores the method variables and their impact. It represents a systemic approach starting by defining the method objectives and critical quality attributes then performing risk assessment and developing experimental design through implementation of a quality strategy and finally managing its lifecycle. Additionally, it implements the recent ICH guidelines Q14. We strongly recommend this concept to be considered in our future work as the implementation of AQbD in hybrid with WAC renders the development of the method easier and the selection of chemicals more accurate. Furthermore, the QbD developed methods are found to be more robust and rugged. Moreover, these methods do not require revalidation due to the lifecycle validation confirming fit for purpose data throughout the whole lifecycle. Briefly, we can say that AQbD provides the right analysis at the right time.

## Conclusion

In the current study, the proposed HPTLC methods serve as simple, economic and sustainable alternative to other multistep complex methods for the concurrent analysis of RMD, FAV and MOL. In the designed methods, hazardous solvents and reagents were superseded by greener alternatives; therefore we expect that our methods represent two attractive tools feasible for implementation in quality control labs for routine pharmaceutical analysis. Moreover, the herein proposed methods were thoroughly evaluated using trichromatic integrative multi-complementary measures by applying the state-of-the-art of metrics. Confirmation of excellent eco-friendliness (greenness) of the methods was carried out using the Eco-Scale, AGREE and the novel MoGAPI tools. Their effective practicality (blueness) was proved by the recent BAGI metric. The superb sustainability (whiteness) of the designed methods was confirmed by the high scores of the RGB 12 algorithm. By integrating excellent analytical performance, tangible eco-compatibility and evident practicality, the HPTLC-NP method excels the HPTLC-RP method in conforming to the concept of fit-for-purpose for application in routine analysis of the three antivirals in quality control labs. This represents a great advantage especially in pharmaceutical companies manufacturing the single drug formulations of the three anti-Covid drugs. This was our main motivator to conduct the presented work.

## Electronic supplementary material

Below is the link to the electronic supplementary material.

Supplementary Material 1

Supplementary Material 2

## Data Availability

All data generated or analyzed during this study are included in this published article and its supplementary information files.
