# Supplementary material for: Comparative study of Normal-phase versus reversed-phase HPTLC methods for the concurrent quantification of three antiviral agents against COVID19: Remdesivir, favipiravir and Molnupiravir: trichromatic sustainability assessment
Source: BMC Chem. 2025 Mar 28;19(1):83. doi: 10.1186/s13065-025-01439-9 (PMC11954355; doi:10.1186/s13065-025-01439-9)
Supplement: Supplementary file 1 — Supplementary Material 1 [file 13065_2025_1439_MOESM1_ESM.pdf]

Supplementary Figures

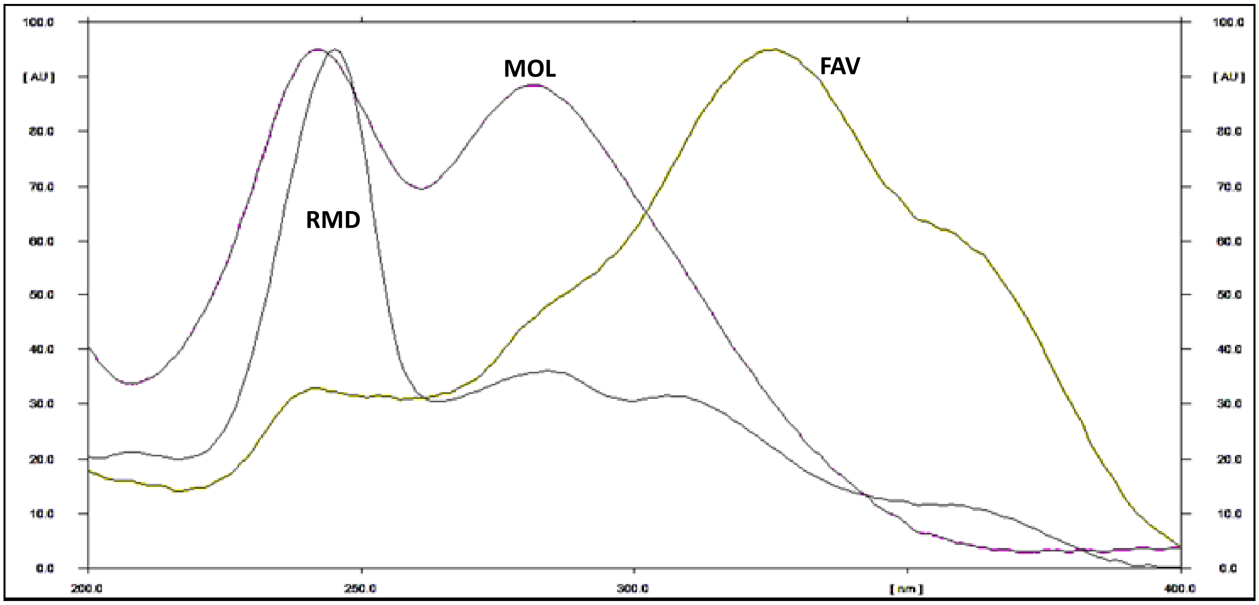

Figure S1: UV spectra of RMD, MOL and FAV.

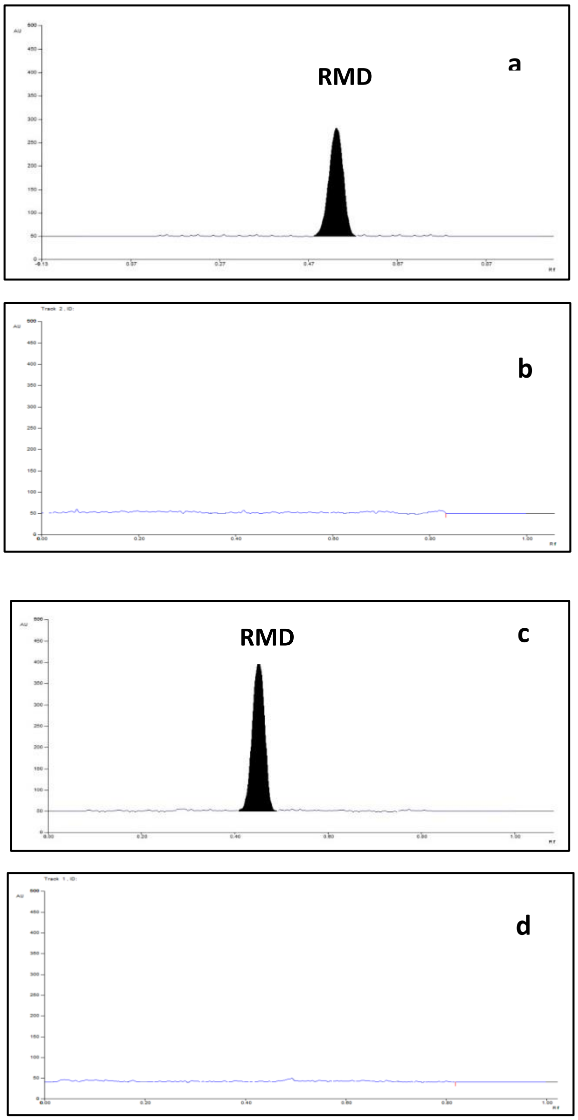

Figure S2: - HPTLC-NP densitograms of analyzed sample of pharmaceutical formulation containing 800 ng/band of RMD (a) and corresponding matrix solution (b) at 244 nm.

- HPTLC-RP densitograms of analyzed sample of pharmaceutical formulation containing 800 ng/band of RMD (c) and corresponding matrix solution (d) at 244 nm.

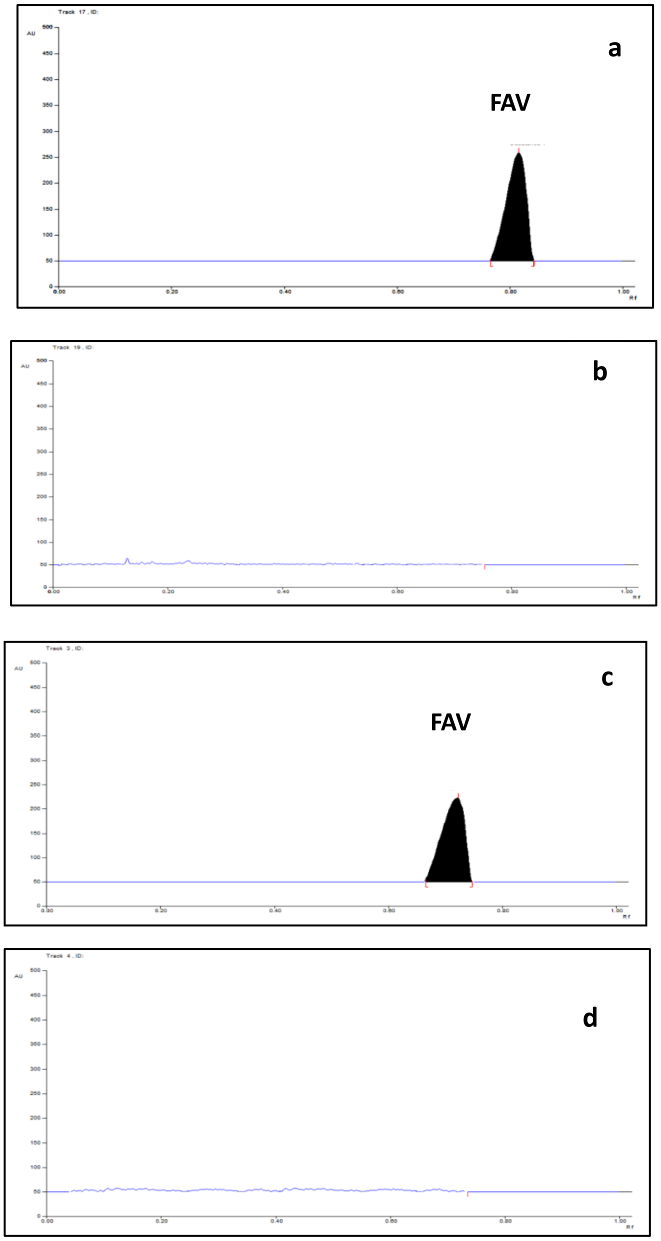

Figure S3: - HPTLC-NP densitograms of analyzed sample of pharmaceutical formulation containing 1000 ng/band of FAV (a) and corresponding matrix solution (b) at 325 nm.
- HPTLC-RP densitograms of analyzed sample of pharmaceutical formulation containing 1000 ng/band of FAV (c) and corresponding matrix solution (d) at 325 nm.

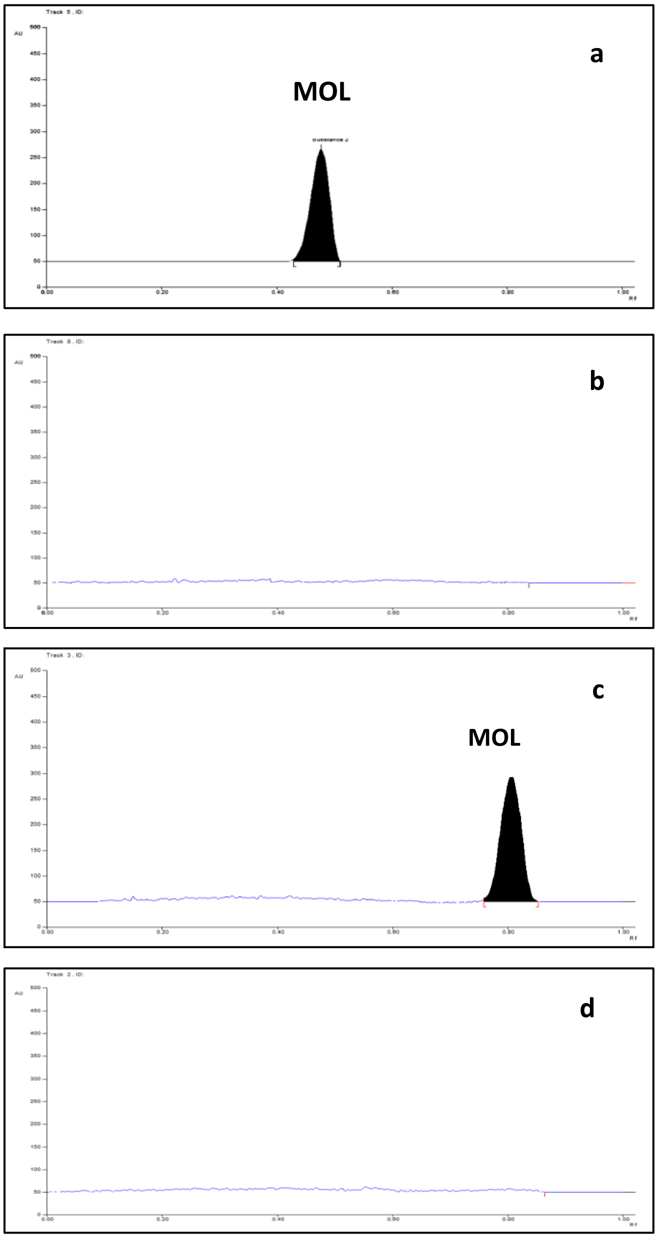

Figure S4: - HPTLC-NP densitograms of analyzed sample of pharmaceutical formulation containing 1000 ng/band of MOL (a) and corresponding matrix solution (b) at 244 nm.

- HPTLC-RP densitograms of analyzed sample of pharmaceutical formulation containing 1000 ng/band of MOL (c) and corresponding matrix solution (d) at 244 nm.

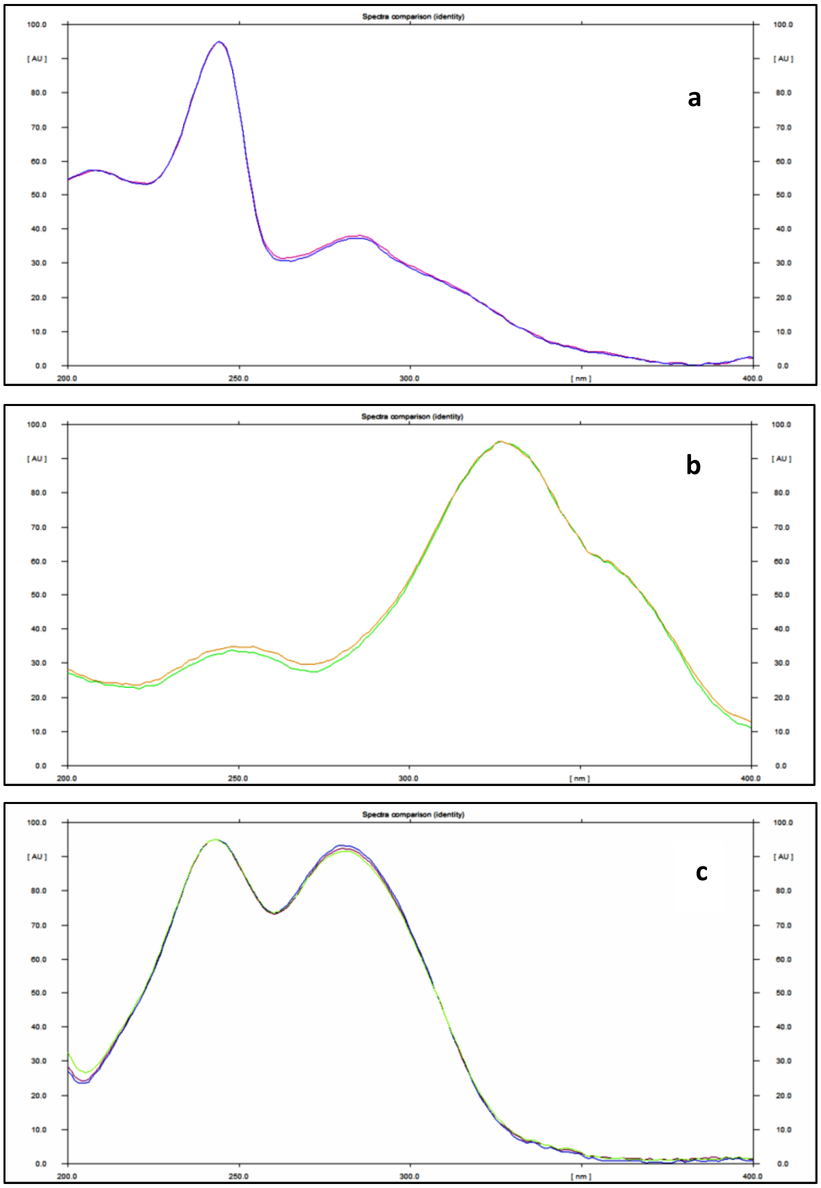

Figure S5a: Peak purity profiling by the HPTLC scanner of UV spectra of standard and sample solutions of RMD (a), FAV (b) and MOL (c) using HPTLC-NP**.**

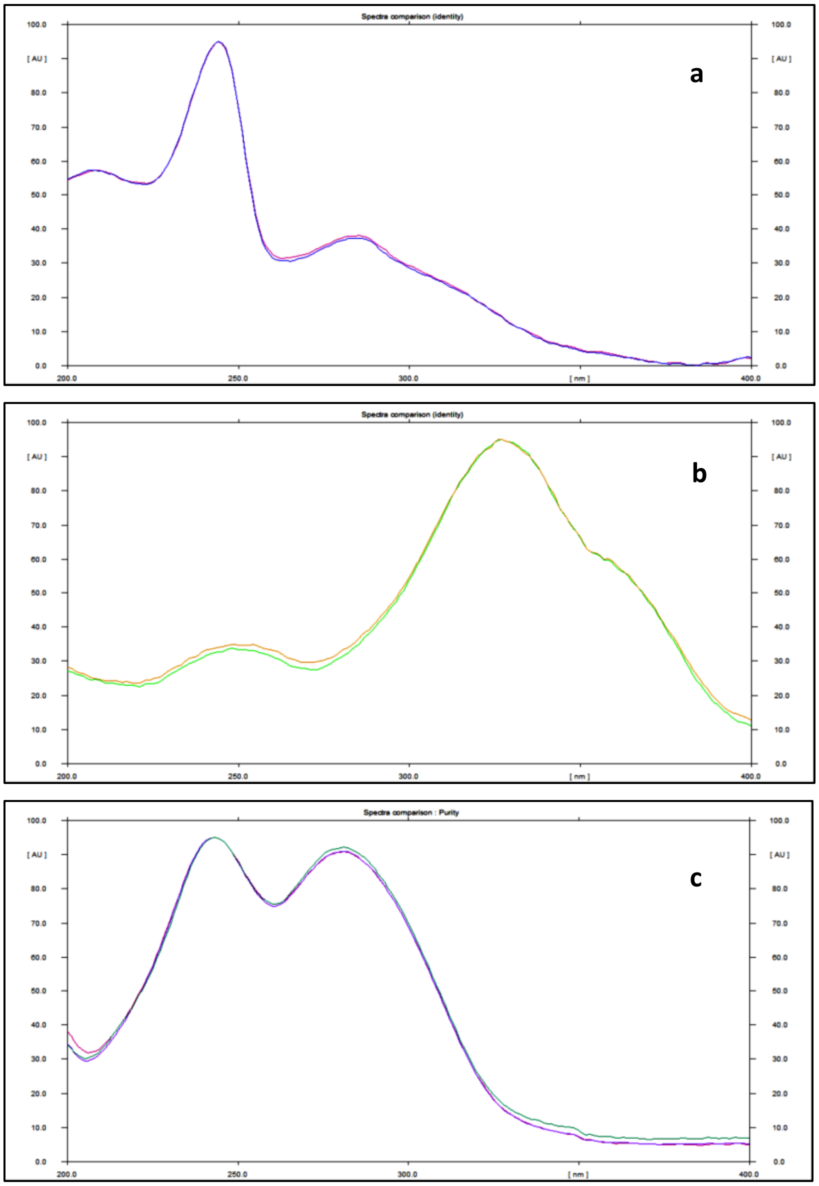

Figure S5b: Peak purity profiling by the HPTLC scanner of UV spectra of standard and sample solutions of RMD (a), FAV (b) and MOL (c) using HPTLC-RP.

Supplementary Tables

Table S1: System suitability parameters for HPTLC-NP and HPTLC-RP methods

Method	HPTLC-NP			HPTLC-RP			Reference Value [46]
Parameter	MOL	RMD	FAV	RMD	FAV	MOL	
R_f	0.47	0.54	0.8	0.46	0.69	0.81	0.1 - 0.9
Symmetry	1.13	1.07	1.06	1.00	1.17	1.1	≈ 1 for symmetrical peak
Tailing factor	1.05	1.11	1.08	1.00	1.08	1.09	0.9 - 1.1
Capacity factor (K')	1.13	0.85	0.25	1.174	0.45	0.23	0.1 – 9
Selectivity (α)	1.32 3.41			2.62 1.92			> 1
Resolution (R_s)	1.30 3.02			3.36 2.5			> 1

aSymmetry factor = b/a, the ratio of back to front width at 10% of peak height.
[b]Tailing factor; T = (a + b)/2 a, at 5% of peak height.
[c]Capacity factor; k´ = (1 - Rf) / Rf.
[d]Selectivity; α = k´1 / k´2.
[e]Resolution; Rs = (Z2-Z1) / 0.5 (W1+W2), where Z is the distance travelled by the analyte from the start line and W is the peak width at 5% of peak height.

Table S2: **Determination of RMD, FAV and MOL laboratory-prepared mixtures using the proposed methods.**

Parameter / Method	Mix no.	RMD				FAV				MOL			
		**Conc.[a]**	**Mean[b] %Rec.**	**RSD%**	**E_r%**	**Conc.[a]**	**Mean[b] %Rec.**	**RSD%**	**E_r%**	**Conc.[a]**	**Mean[b] %Rec**	**RSD%**	**E_r%**
**HPTLC-NP**	**1**	**800**	100.35	0.33	0.35	**800**	100.39	0.32	0.39	**800**	99.92	0.46	-0.08
	**2**	**600**	100.03	0.16	0.03	**1200**	100.08	0.68	0.08	**600**	99.58	0.41	-0.42
	**3**	**500**	100.13	0.58	0.13	**1000**	100.43	0.32	0.43	**1000**	100.13	0.49	0.13
	**4**	**800**	99.88	0.59	-0.12	**400**	100.14	0.31	0.14	**400**	100.19	0.35	0.19
	**5**	**400**	99.97	0.73	-0.03	**400**	100.11	0.75	0.11	**800**	99.65	0.60	-0.35
**HPTLC-RP**	**1**	**800**	99.99	0.56	-0.01	**800**	100.16	0.68	0.16	**800**	100.25	0.23	0.25
	**2**	**600**	99.96	0.66	-0.04	**1200**	99.66	0.49	-0.34	**600**	100.44	0.47	0.44
	**3**	**500**	99.92	0.44	-0.08	**1000**	99.84	0.71	-0.16	**1000**	100.02	0.07	0.02
	**4**	**800**	100.33	0.74	0.33	**400**	100.04	0.75	0.04	**400**	100.48	0.38	0.48
	**5**	**400**	99.43	0.44	-0.57	**400**	99.95	0.27	-0.05	**800**	100.08	0.50	0.08

[a] Concentration unit (ng/band).
[b] Mean of three determinations

Table S3: Robustness study of the proposed HPTLC-NP method

Parameters	Drug	Conc. ng/band (μg/mL)	Peak area ± SD	RSD%	R_f ± SD
**Working wavelength ± 2 nm**	**RMD**	600 (120)	4872 ± 26.14	0.54	0.54 ± 0.006
	**FAV**	1200 (240)	9753 ± 33.34	0.34	0.81 ± 0.006
	**MOL**	600 (240)	3103 ± 13.06	0.42	0.47 ± 0.006
**Mobile phase composition***	**RMD**	600 (120)	4883 ± 22.97	0.47	0.53 ± 0.006
	**FAV**	1200 (240)	9796 ± 41.25	0.42	0.81 ± 0.008
	**MOL**	600 (240)	3166 ± 12.55	0.40	0.46 ± 0.008
**Mobile phase volume 15 mL ± 2mL**	**RMD**	600 (120)	4888 ± 32.29	0.66	0.54 ± 0.004
	**FAV**	1200 (240)	9835 ± 42.07	0.43	0.81 ± 0.006
	**MOL**	600 (120)	3197 ± 27.05	0.85	0.45 ± 0.002
**Time from chromatography to scan (0, 20, 40, 60 min)**	**RMD**	600 (120)	5002 ± 17.35	0.35	0.54 ± 0.006
	**FAV**	1200 (240)	9877 ± 26.01	0.26	0.80 ± 0.010
	**MOL**	600 (120)	3158 ± 26.17	0.83	0.45 ± 0.006
**Duration of saturation 15 min ± 5min**	**RMD**	600 (120)	4986 ± 16.99	0.34	0.54 ± 0.005
	**FAV**	1200 (240)	9987 ± 98.42	0.99	0.81 ± 0.006
	**MOL**	600 (120)	3148 ± 41.00	1.30	0.45 ± 0.006

***(Ethyl acetate: ethanol: water), (94 : 4 : 2.5, v/v), (84 : 4: 2.5, v/v), (104 : 4 : 2.5, v/v)**

Table S4: Robustness study of the proposed HPTLC-RP method

Parameters	Drug	Conc. ng/band (μg/mL)	Peak area ± SD	RSD%	R_f ± SD
**Working wavelength ± 2 nm**	**RMD**	600 (120)	7164 ± 19.56	0.27	0.46 ± 0.00
	**FAV**	1200 (240)	8401 ± 79.49	0.95	0.69 ± 0.00
	**MOL**	600 (240)	4201 ± 54.13	1.29	0.81 ± 0.00
**Mobile phase composition***	**RMD**	600 (120)	7124 ± 51.42	0.72	0.47 ± 0.015
	**FAV**	1200 (240)	8579 ± 193.94	2.26	0.70 ± 0.010
	**MOL**	600 (240)	4128 ± 88.92	2.15	0.80 ± 0.006
**Mobile phase volume 25 mL ± 2mL**	**RMD**	600 (120)	7147 ± 47.21	0.66	0.46 ± 0.006
	**FAV**	1200 (240)	8534 ± 36.51	0.43	0.69 ± 0.006
	**MOL**	600 (120)	4199 ± 5.25	0.13	0.81 ± 0.006
**Time from chromatography to scan (0, 20, 40, 60 min)**	**RMD**	600 (120)	7185 ± 59.28	0.83	0.47 ± 0.010
	**FAV**	1200 (240)	8497 ± 80.15	0.94	0.69 ± 0.008
	**MOL**	600 (120)	4214 ± 49.66	1.18	0.81 ± 0.006
**Duration of saturation 35 min ± 5 min**	**RMD**	600 (120)	7172 ±75.92	1.06	0.47 ±0.005
	**FAV**	1200 (240)	8580 ± 76.16	0.89	0.71 ± 0.021
	**MOL**	600 (120)	4220 ± 23.93	0.57	0.81 ± 1.24

***(ethanol: water), (55: 40), (60: 40) and (65: 40).**

Table S5: Application of the proposed HPTLC methods for the analysis of cited drugs in their pharmaceutical dosage forms and comparison with reference methods [19, 32]

Drug	Method	% Recovery ± SD[a]	RSD%[b]
RMD	I	99.79 ± 0.92	0.92
	II	100.13 ± 1.09	1.09
	Reference [32]	100.23 ± 1.90	1.90
	Single factor ANOVA: F = 0.143, F (critical) = 3.885		
FAV	I	99.93 ± 0.97	0.97
	II	100.28 ± 1.04	1.04
	Reference [32]	99.60 ± 1.21	1.21
	Reference [19]	99.71 ± 1.25	1.25
	Single factor ANOVA: F = 0.356, F (critical) = 3.239		
MOL	I	100.31 ± 0.92	0.92
	II	99.18 ± 0.74	0.75
	Reference [19]	99.53 ± 1.07	1.08
	Single factor ANOVA: F = 1.957, F (critical) = 3.885		

[a] Mean ± standard deviation for five determinations.

[b] % Relative standard deviation.

Table S6: Application of the proposed HPTLC methods for the analysis of cited drugs in their pharmaceutical dosage forms using standard addition method

Drug	Method	% Recovery ± SD[a]	RSD%[b]
RMD	I	100.34 ± 0.77	0.77
	II	100.04 ± 1.19	1.19
FAV	I	100.12 ± 1.42	1.42
	II	99.78 ± 1.35	1.35
MOL	I	99.30 ± 0.94	0.95
	II	100.50 ± 0.82	0.82

[a] Mean ± standard deviation for five determinations.

[b] % Relative standard deviation.

Table S7: The penalty points of the proposed HPTLC methods and reported HPLC-HRMS method according to the Analytical Eco-scale

Reagents/ Instruments	Penalty Points (PPs)		
	HPTLC-NP	HPTLC-RP	HPLC-HRMS [16]
**Ethyl acetate**	4	-	
**Absolute ethanol**	4	4	
**Acetonitrile**			4
**Formic acid**			6
**Methanol**			6
**TLC**	1	1	
**LC-MS**			2
**Occupational hazards**	0	0	3
**Waste**	1	3	3
**PPs**	**10**	**8**	**24**
**Eco-scale score**	**90**	**92**	**76**

Table S8: Whiteness assessment of the proposed HPTLC methods and reported HPLC-HRMS method using RGB 12 algorithm

Method	R (%)	G (%)	B (%)	Whiteness (%)
**HPTLC-NP**	87.5	97.5	102.1	**95.7**
**HPTLC-RP**	90.0	98.3	93.3	**93.9**
**HPLC-HRMS [16]**	100.0	80.8	75.0	**85.3**
