# Supplementary material for: Comparative study of Normal-phase versus reversed-phase HPTLC methods for the concurrent quantification of three antiviral agents against COVID19: Remdesivir, favipiravir and Molnupiravir: trichromatic sustainability assessment
Source: BMC Chem. 2025 Mar 28;19(1):83. doi: 10.1186/s13065-025-01439-9 (PMC11954355; doi:10.1186/s13065-025-01439-9)

Supplementary File-2

MoGAPI Input Data

Modified Green Analytical Procedure Index

HPTLC-NP Method

MoGAPI input data

**SAMPLE PREPARATION**	
**1 - Collection:**	On-line or at-line
**2 - Preservation:**	None
**3 - Transport:**	None
**4 - Storage:**	Under normal conditions
**5 - Type of method:**	Simple procedures, e.g., filtration, c
**6 - Scale of extraction:**	Micro-extraction
**7 - Solvents/reagents used:**	Green solvents/reagents used
**8 - Additional treatment:**	None
**REAGENT AND SOLVENTS**	
**9 - Amount:**	< 10 mL (< 10 g)
**10 - Health hazard:**	Moderately toxic; could cause temp
**11 - Safety hazard:**	Highest NFPA flammability or instal
**INSTRUMENTATION**	
**12 - Energy:**	≤1.5 kWh per sample
**13 - Occupational hazard:**	Hermetic sealing of analytical proce
**14 - Waste:**	< 1 mL (< 1 g)
**15 - Waste treatment:**	No treatment
**16 - QUANTIFICATION:**	Yes

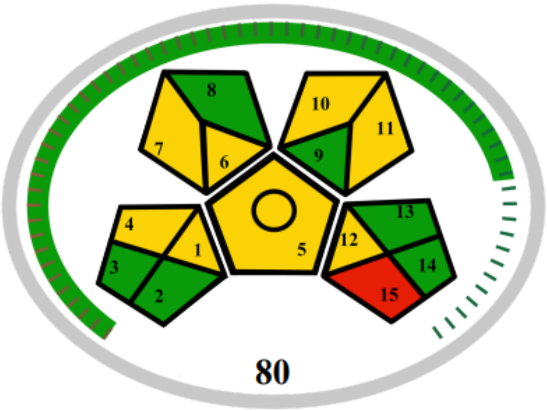

HPTLC-RP Method

MoGAPI input data

**SAMPLE PREPARATION**	
**1 - Collection:**	On-line or at-line
**2 - Preservation:**	None
**3 - Transport:**	None
**4 - Storage:**	Under normal conditions
**5 - Type of method:**	Simple procedures, e.g., filtration, c
**6 - Scale of extraction:**	Micro-extraction
**7 - Solvents/reagents used:**	Green solvents/reagents used
**8 - Additional treatment:**	None
**REAGENT AND SOLVENTS**	
**9 - Amount:**	< 10 mL (< 10 g)
**10 - Health hazard:**	Moderately toxic; could cause temp
**11 - Safety hazard:**	Highest NFPA flammability or instal
**INSTRUMENTATION**	
**12 - Energy:**	≤1.5 kWh per sample
**13 - Occupational hazard:**	Hermetic sealing of analytical proce
**14 - Waste:**	1–10 mL (1–10 g)
**15 - Waste treatment:**	No treatment
**16 - QUANTIFICATION:**	Yes

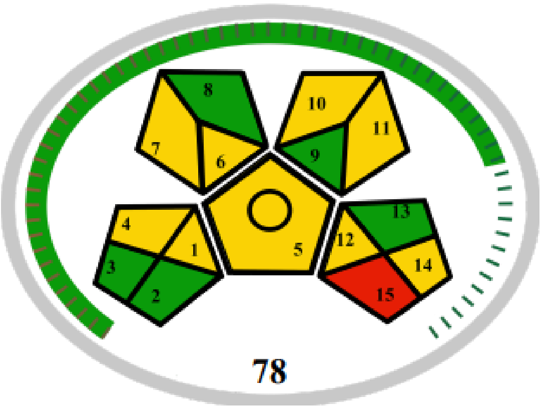

HPLC-HRMS Method [16]

MoGAPI input data

**SAMPLE PREPARATION**	
**1 - Collection:**	Off-line
**2 - Preservation:**	None
**3 - Transport:**	None
**4 - Storage:**	Under normal conditions
**5 - Type of method:**	Extraction required
**6 - Scale of extraction:**	Nano-extraction
**7 - Solvents/reagents used:**	Non-green solvents/reagents used
**8 - Additional treatment:**	Simple treatments (clean up, solver
**REAGENT AND SOLVENTS**	
**9 - Amount:**	< 10 mL (< 10 g)
**10 - Health hazard:**	Moderately toxic; could cause temp
**11 - Safety hazard:**	Highest NFPA flammability or instat
**INSTRUMENTATION**	
**12 - Energy:**	> 1.5 kWh per sample
**13 - Occupational hazard:**	Hermetic sealing of analytical proce
**14 - Waste:**	1–10 mL (1–10 g)
**15 - Waste treatment:**	No treatment
**16 - QUANTIFICATION:**	Yes

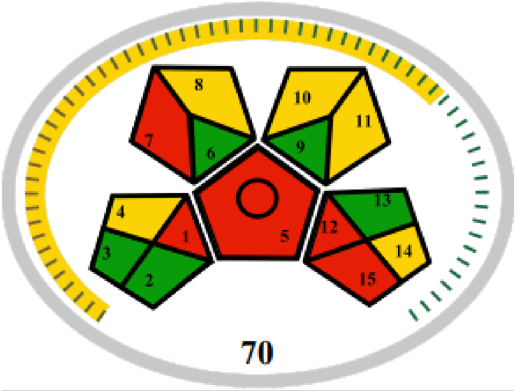

AGREE Input Data

Analytical Greenness Report Sheets

HPTLC-NP Method

AGREE Report Sheet

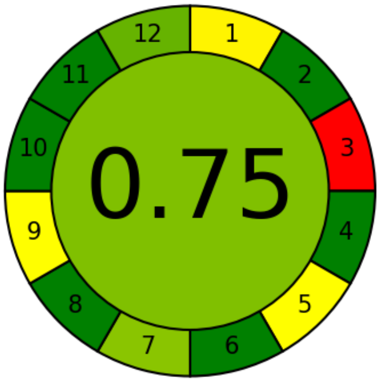

1. Sample treatment
2. Sample amount
3. Device positioning
4. Sample prep. stages
5. Automation, miniaturization
6. Derivatization
7. Waste
8. Analysis throughput
9. Energy consumption
10. Source of reagents
11. Toxicity
12. Operator's safety

Criteria	Score	Weight
1. Direct analytical techniques should be applied to avoid sample treatment.	0.48	2
2. Minimal sample size and minimal number of samples are goals.	1.0	2
3. If possible, measurements should be performed in situ.	0.0	2
4. Integration of analytical processes and operations saves energy and reduces the use of reagents.	1.0	2
5. Automated and miniaturized methods should be selected.	0.5	2
6. Derivatization should be avoided.	1.0	2
7. Generation of a large volume of analytical waste should be avoided, and proper management of analytical waste should be provided.	0.73	2
8. Multi-analyte or multi-parameter methods are preferred versus methods using one analyte at a time.	1.0	2
9. The use of energy should be minimized.	0.5	2
10. Reagents obtained from renewable sources should be preferred.	1.0	2
11. Toxic reagents should be eliminated or replaced.	1.0	2
12. Operator's safety should be increased.	0.8	2

HPTLC-RP Method

AGREE Report Sheet

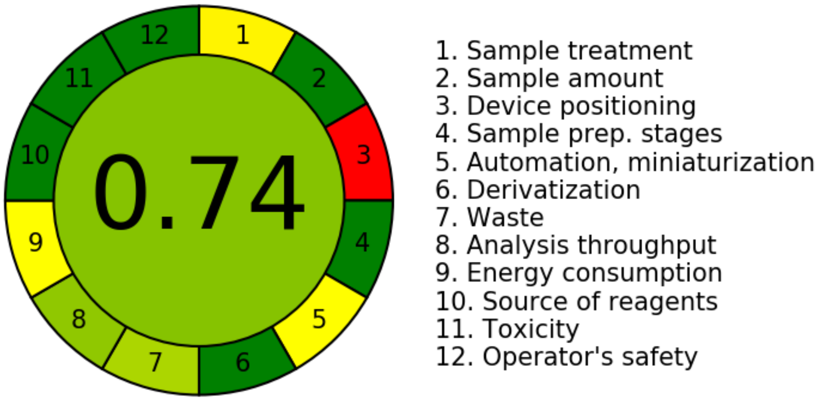

Criteria	Score	Weight
1. Direct analytical techniques should be applied to avoid sample treatment.	0.48	2
2. Minimal sample size and minimal number of samples are goals.	1.0	2
3. If possible, measurements should be performed in situ.	0.0	2
4. Integration of analytical processes and operations saves energy and reduces the use of reagents.	1.0	2
5. Automated and miniaturized methods should be selected.	0.5	2
6. Derivatization should be avoided.	1.0	2
7. Generation of a large volume of analytical waste should be avoided, and proper management of analytical waste should be provided.	0.66	2
8. Multi-analyte or multi-parameter methods are preferred versus methods using one analyte at a time.	0.72	2
9. The use of energy should be minimized.	0.5	2
10. Reagents obtained from renewable sources should be preferred.	1.0	2
11. Toxic reagents should be eliminated or replaced.	1.0	2
12. Operator's safety should be increased.	1.0	2

HPLC-HRMS Method [16]

AGREE Report Sheet

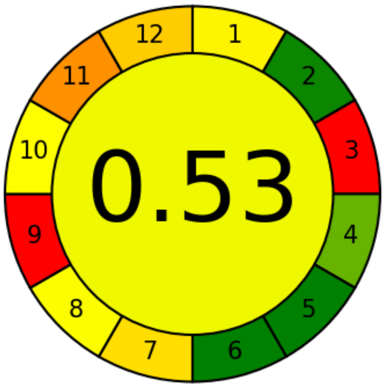

1. Sample treatment
2. Sample amount
3. Device positioning
4. Sample prep. stages
5. Automation, miniaturization
6. Derivatization
7. Waste
8. Analysis throughput
9. Energy consumption
10. Source of reagents
11. Toxicity
12. Operator's safety

Criteria	Score	Weight
1. Direct analytical techniques should be applied to avoid sample treatment.	0.48	2
2. Minimal sample size and minimal number of samples are goals.	0.98	2
3. If possible, measurements should be performed in situ.	0.0	2
4. Integration of analytical processes and operations saves energy and reduces the use of reagents.	0.8	2
5. Automated and miniaturized methods should be selected.	1.0	2
6. Derivatization should be avoided.	1.0	2
7. Generation of a large volume of analytical waste should be avoided, and proper management of analytical waste should be provided.	0.44	2
8. Multi-analyte or multi-parameter methods are preferred versus methods using one analyte at a time.	0.51	2
9. The use of energy should be minimized.	0.0	2
10. Reagents obtained from renewable sources should be preferred.	0.5	2
11. Toxic reagents should be eliminated or replaced.	0.28	2
12. Operator's safety should be increased.	0.4	2

Blue Applicability Grade Index

BAGI Input Data

HPTLC-NP Method

BAGI Input Data

1. Type of analysis	Quantitative and confirmatory ▾
2. Multi- or single-element analysis	Multi-element analysis for 6-15 compounds of the s ▾
3. Analytical technique	Simple instrumentation available in most labs (UV, H ▾
4. Simultaneous sample preparation	1 ▾
5. Sample preparation	Not required or on-site sample preparation if require ▾
6. Samples per h	>10 ▾
7. Reagents and materials	Common commercially available reagents (methanc ▾
8. Preconcentration	No preconcentration required. Required sensitivity a ▾
9. Degree of automation	Semi-automated with common devices (e.g. HPLC a ▾
10. Amount of sample	<100 µL (or mg) bioanalytical samples; <10 mL (or g) food/environmen ▾

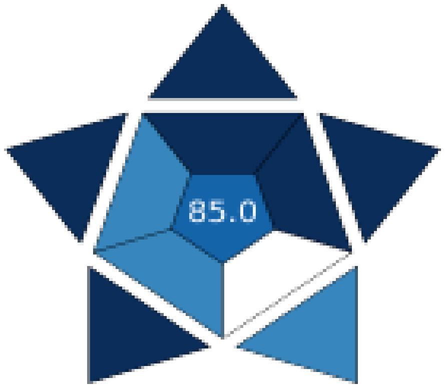

HPTLC-RP Method

BAGI Input Data

1. Type of analysis	Quantitative and confirmatory
2. Multi- or single-element analysis	Multi-element analysis for 6-15 compounds of the s
3. Analytical technique	Simple instrumentation available in most labs (UV, H
4. Simultaneous sample preparation	1
5. Sample preparation	Not required or on-site sample preparation if require
6. Samples per h	5-10
7. Reagents and materials	Common commercially available reagents (methan
8. Preconcentration	No preconcentration required. Required sensitivity a
9. Degree of automation	Semi-automated with common devices (e.g. HPLC a
10. Amount of sample	<100 µL (or mg) bioanalytical samples; <10 mL (or g) food/environmen

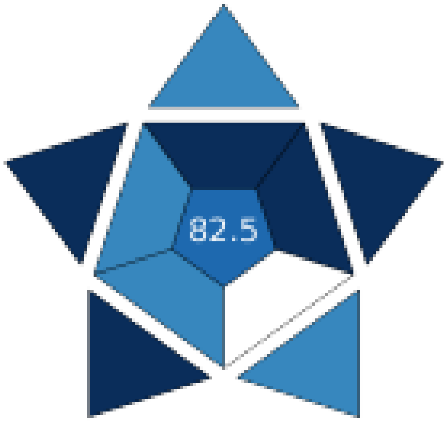

HPLC-HRMS Method [16]

BAGI Input Data

1. Type of analysis	Quantitative and confirmatory ▾
2. Multi- or single-element analysis	Multi-element analysis for 6-15 compounds of the s ▾
3. Analytical technique	Instrumentation that is not commonly available in m ▾
4. Simultaneous sample preparation	1 ▾
5. Sample preparation	Miniaturized extraction sample preparation (SPME, ▾
6. Samples per h	2-4 ▾
7. Reagents and materials	Common commercially available reagents (methanc ▾
8. Preconcentration	Preconcentration required. Required sensitivity is m ▾
9. Degree of automation	Semi-automated with common devices (e.g. HPLC a ▾
10. Amount of sample	<100 µL (or mg) bioanalytical samples; <10 mL (or g) food/environmen ▾

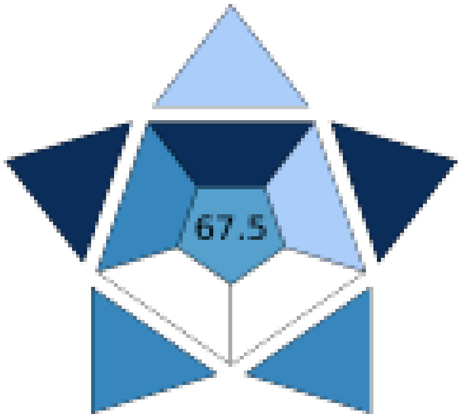

Supplement: Supplementary file 2 — Supplementary Material 2 [file 13065_2025_1439_MOESM2_ESM.pdf]
